# Advanced Carcinoid Tricuspid Valve Disease Associated with Delayed Recognition and Loss of the Interventional Window in a Patient with Ileal Neuroendocrine Tumor: A Case Report

**DOI:** 10.3390/diagnostics16193160

**Published:** 2026-09-29

**Authors:** Ionela Cotfas, Cosmin Banceu, Horațiu Suciu, Klara Brînzaniuc, Ileana Voichița Sirbu

**Affiliations:** 1 Department of Morphological Sciences, George Emil Palade University of Medicine, Pharmacy, Science and Technology of Târgu Mureș, Gheorghe Marinescu Street 38, 540139 Târgu Mureș, Romania; 2 Department of Clinical and Medical-Surgical Disciplines, George Emil Palade University of Medicine, Pharmacy, Science and Technology of Târgu Mureș, Gheorghe Marinescu Street 38, 540139 Târgu Mureș, Romania; 3 Emergency Institute for Cardiovascular Diseases and Transplantation (IUBCVT), Gheorghe Marinescu Street 50, 540136 Târgu Mureș, Romania

**Keywords:** carcinoid heart disease, neuroendocrine tumor, carcinoid syndrome, tricuspid regurgitation, echocardiographic surveillance, coaptation gap, transcatheter edge-to-edge repair, missed therapeutic window

## Abstract

**Background and Clinical Significance:** Carcinoid heart disease remains one of the most serious complications of carcinoid syndrome, yet cardiac involvement may still be recognized late, when valvular damage is already advanced and treatment options are limited. **Case Presentation:** We report a 70-year-old man with a well-differentiated ileal neuroendocrine tumor (NET G2, Ki-67 up to 5%), diagnosed in December 2019. Over six years, the disease progressed with liver metastases, markedly rising 24 h urinary 5-HIAA levels and clinically active carcinoid syndrome, and was treated sequentially with somatostatin analogues, everolimus and ^177^Lu-DOTATOC. Throughout this period, oncological evaluation was frequent, whereas structured cardiac assessment was essentially absent until 2025. When the patient was referred for evaluation for transcatheter tricuspid repair, echocardiography demonstrated advanced carcinoid tricuspid valve disease with severe regurgitation, mild stenosis, marked leaflet thickening and tethering, and a coaptation gap of 17 mm—far beyond the ~7 mm threshold associated with favorable edge-to-edge repair and the >10 mm range associated with poor outcome. Because of the advanced valve remodeling and the overall clinical burden, the patient was not a candidate for transcatheter edge-to-edge repair or surgery and subsequently died. **Conclusions:** This case illustrates how carcinoid heart disease can progress beyond correctability and supports regular echocardiographic surveillance in patients with carcinoid syndrome, so that valve involvement is identified while intervention is still anatomically feasible.

## 1. Introduction

Neuroendocrine tumors (NETs), particularly those arising from the small intestine and ileum, are uncommon but increasingly diagnosed malignancies, with registry data reporting an incidence of approximately 3–5 cases per 100,000 persons annually [1,2]. Diagnosis is often delayed by the indolent course of the disease, and many patients already have hepatic metastases at the time of confirmation. Carcinoid syndrome is a clinically significant complication of metastatic NET, resulting from systemic exposure to vasoactive substances such as serotonin and tachykinins. Within the cardiovascular system, prolonged exposure to NET-derived mediators can lead to carcinoid heart disease (CHD), characterized by fibrotic endocardial deposits on the endocardial surface and on the valvular structures [3,4,5].

Valvular involvement in carcinoid syndrome predominantly affects the right-sided valves, the tricuspid valve being the most frequently and most severely involved, followed by the pulmonary valve. Carcinoid disease of the tricuspid valve is characterized by fibrous remodeling of the leaflets with progressive thickening, leading to retraction, restricted systolic–diastolic motion and defective coaptation. Clinically, these alterations manifest as varying degrees of valvular regurgitation, stenosis, or both [6,7].

Early detection is essential, as surgical and interventional therapies performed within the optimal therapeutic window have a substantially higher likelihood of success. Despite guideline endorsement of routine echocardiographic screening in patients with carcinoid syndrome, implementation in clinical practice remains variable, and inconsistent, and cardiac involvement is frequently recognized late [8]. Delayed diagnosis of tricuspid valve involvement is often associated with advanced structural damage and global heart failure, with high surgical and interventional risk and progressively worsening prognosis [9,10].

This case report underlines the importance of cardiovascular vigilance in identifying the cardiac consequences of carcinoid disease at a stage that remains amenable to therapy. A timeline of the oncological and cardiac course is provided in Table 1.

## 2. Case Presentation

A 70-year-old male patient was diagnosed in December 2019 with a well-differentiated neuroendocrine tumor (NET G2, Ki-67 up to 5%, pT4 pN1 cM1) located at the ileal level. The diagnosis followed intestinal surgery for a stenosing small-bowel lesion causing obstruction, with segmental ileal and mesenteric lymph node resection. Histopathology showed complete chromogranin A and synaptophysin expression, lymphovascular emboli, nodal metastasis and perineural invasion.

The disease progressed with hepatic metastases and active carcinoid syndrome (diarrhea, flushing, weight loss). Serotonin overproduction was documented by 24 h urinary 5-HIAA, which rose markedly over the disease course (2 mg/24 h in 2020; 5.3 mg/24 h in early 2021; 110 mg/24 h in September 2023; reference range 2–9 mg/24 h), paralleled by rising chromogranin A. The patient was treated with octreotide (from July 2020), followed by lanreotide (February–August 2021, paused after symptom resolution). Following symptomatic and radiological recurrence in 2023, lanreotide was resumed, then switched to octreotide and discontinued because of side effects; everolimus was administered from August 2024 to April 2025. In May 2025, first ^177^Lu-DOTATOC peptide receptor radionuclide therapy (7.5 GBq) was undertaken for increasing hepatic tumor burden; imaging demonstrated somatostatin receptor-positive liver metastases, retroperitoneal nodal uptake indicating nodal metastatic disease, and diffuse peritoneal haziness with small nodular thickening and minimal ascites suggestive of peritoneal carcinosis.

Throughout the earlier disease course, management remained essentially oncological, and structured cardiac surveillance was not specifically recommended; no scheduled echocardiographic follow-up was arranged despite progressive hepatic metastases and rising 5-HIAA. Cardiac assessment was undertaken only once cardiac symptoms had developed (Table 1).

The first documented cardiac note (May 2025) described moderate tricuspid regurgitation under cardiological surveillance. Later in 2025, transthoracic echocardiography documented NYHA class II heart failure with reduced ejection fraction (LVEF 36%), severe primary tricuspid regurgitation, moderate aortic regurgitation on a tricuspid aortic valve, mild mitral regurgitation, and a patent foramen ovale.

Cardiac magnetic resonance imaging (MRI) performed in December 2025 showed a non-dilated left ventricle with severe global systolic dysfunction (LVEF 26%; LV end-diastolic volume 108 mL), diffuse hypokinesia and no myocardial edema, fibrosis or late gadolinium enhancement (ECV 24%). Right ventricular systolic function was preserved. There was severe tricuspid regurgitation (calculated regurgitant fraction 51%), right atrial dilatation and an interatrial septal aneurysm.

In February 2026, the patient was referred to our center for evaluation for transcatheter tricuspid edge-to-edge repair (T-TEER). He reported worsening dyspnea, with an NT-proBNP of 4322 pg/mL, mild macrocytic anemia, mild thrombocytopenia and a cholestatic pattern of liver injury. The New York Heart Association (NYHA) functional class was IV at this presentation.

Transthoracic echocardiography in our laboratory showed mixed carcinoid tricuspid valve disease with severe regurgitation and mild stenosis. The valvular leaflets appeared thickened and tethered, with severely restricted systolic–diastolic motion and a coaptation gap of 17 mm between the septal and anterior cusps, producing an infiltrated, tube-like appearance of the tricuspid apparatus. The regurgitation was graded as severe on comprehensive echocardiographic analysis (vena contracta 12 mm, PISA radius 0.9 cm, EROA 0.6 cm^2^, right ventricular–right atrial gradient 47 mmHg). A concomitant stenotic component was present, with a mean transvalvular gradient of 6.2/3.1 mmHg (peak/mean), and three-dimensional flexi-slice reconstruction showed a planimetric orifice area of 1.7 cm^2^. The pulmonary valve presented mild-to-moderate regurgitation (Figure 1).

The right ventricle was enlarged, measuring 42 mm in subtricuspid diameter (RV > LV), 34 mm mid-ventricular and 73 mm longitudinal (right-ventricle-focused apical four-chamber view). Longitudinal right ventricular function was preserved, with a tricuspid annular plane systolic excursion (TAPSE) of 24 mm, a tricuspid lateral annular systolic velocity (S′) of 14 cm/s and a fractional area change (FAC) of 40%. The right atrium was enlarged (81 mL) and presented a protruding Eustachian valve, with grade II spontaneous echo contrast. The interatrial septum was aneurysmal, with a patent foramen ovale and right-to-left shunting; together with the prominent Eustachian valve and grade II spontaneous echo contrast, these findings indicated elevated right-heart pressures and a right-to-left shunt pathway of potential relevance for paradoxical embolism and for any future transcatheter access planning.

Regarding the left cardiac chambers and valves, the left ventricle had normal dimensions, with interventricular septal flattening visible in the parasternal views. A calculated ejection fraction of 37% (EDV 93 mL, ESV 58 mL) was measured with the Simpson biplane method; three-dimensional volumetric assessment was not feasible because of suboptimal acoustic windows. The left atrium had a normal volume. The aortic valve was tricuspid, showed preserved systolic opening with associated moderate-to-severe aortic regurgitation (PHT 262 ms, EROA 0.3 cm^2^, regurgitant volume 46 mL), in the absence of dilatation of the aortic root or ascending aorta. Holter monitoring showed sinus rhythm with frequent isolated ventricular and supraventricular ectopy and no malignant arrhythmia.

The mitral valve presented thickened leaflets with one hypermobile chord attached to the anterior leaflet, and focal posterior annular calcification, resulting in mild mitral regurgitation without a significant transvalvular gradient.

The echocardiographic probability of pulmonary hypertension was assessed as high on the basis of interventricular septal flattening, right ventricular and atrial enlargement, and an estimated pulmonary artery systolic pressure of ~50 mmHg; in the setting of severe tricuspid regurgitation, Doppler-derived pulmonary pressure estimates are less reliable, and right-heart catheterization was not performed, so this remained a non-invasive assessment.

Given the advanced carcinoid remodeling of the tricuspid valve, the large coaptation gap and the mixed lesion, the patient was considered unsuitable for transcatheter edge-to-edge repair. Following a multidisciplinary discussion involving cardiology, cardiac surgery and oncology (rather than a formal structured Heart Team meeting), surgical valve replacement was also judged inappropriate. The high TriScore (estimated ~48% in-hospital mortality) was driven principally by advanced NYHA class, clinical signs of right heart failure and high-dose loop diuretic requirement, together with reduced left ventricular ejection fraction and elevated bilirubin; right ventricular longitudinal function was preserved (TAPSE 24 mm, FAC 40%). Although EuroSCORE II estimated a lower operative mortality (4.8%), the disease-specific TriScore, together with peritoneal carcinosis, NYHA class IV heart failure and limited oncological prognosis, placed the patient in a prohibitive-risk category [11]. No transcatheter or surgical option remained feasible. The patient died later in 2026, within several months of this evaluation; the exact date could not be confirmed from available records.

## 3. Discussion

Carcinoid heart disease is one of the most significant cardiovascular complications of carcinoid syndrome, predominantly involving the right-sided valves in patients with long-standing exposure to vasoactive mediators. The central point of this case is not the presence of severe tricuspid disease itself, but that cardiac involvement was recognized late and was not followed in a structured manner. In this patient, the absence of cardiac surveillance did not reflect patient non-adherence but rather the lack of a structured multidisciplinary pathway [12]: cardiac monitoring was not incorporated into oncological follow-up, and echocardiography was prompted only by symptoms. Current guidance recommends screening with 5-HIAA, natriuretic peptides and transthoracic echocardiography, with follow-up intervals adapted to established cardiac involvement [6,9,13].

This case supports regarding echocardiography not only as a diagnostic tool but as a means of determining therapeutic timing. Serial echocardiography characterizes leaflet thickening and retraction, right-heart remodeling, pulmonary valve involvement and the structural feasibility of intervention. Biochemical burden, and 5-HIAA in particular, correlates with echocardiographic progression and mortality [14,15]. In our patient, 24 h urinary 5-HIAA rose more than tenfold above the upper reference limit over the disease course, a trajectory that would ordinarily prompt close cardiac monitoring.

The assessment of left ventricular systolic function illustrated a recognized imaging challenge: echocardiography (LVEF 37%, February 2026) and cardiac MRI (LVEF 26%, December 2025) yielded different estimates. Rather than indicating that either technique is inaccurate, this difference is best understood as a consequence of ventricular interdependence and methodological differences between modalities, compounded by the two-month interval and differing loading conditions. Cardiac MRI remains the reference standard for ventricular volumes; two-dimensional echocardiography tends to underestimate ventricular volumes and consequently overestimate LVEF, a pattern well described when comparing the two modalities and in volume-overload states [16,17]. In severe right ventricular overload, septal displacement toward the left ventricle further challenges the geometric assumptions of both techniques, and suboptimal acoustic windows precluded three-dimensional volumetric analysis. These estimates are therefore best interpreted together rather than in isolation.

Although the definitive treatment for severe carcinoid tricuspid regurgitation remains surgical valve replacement, our patient was at prohibitive risk and had a high overall clinical burden, including peritoneal carcinosis and NYHA class IV heart failure. Transcatheter tricuspid valve replacement (TTVR) may be considered in patients excluded from both surgery and edge-to-edge repair. An ongoing prospective study [18] is evaluating a custom transcatheter tricuspid valved stent graft in carcinoid disease; notably, that device is a heterotopic bicaval valved stent graft, which addresses venous congestion rather than the tricuspid valve anatomy itself, so the leaflet remodeling and coaptation gap that precluded T-TEER would not, in themselves, have been the limiting factors for such a device. Nonetheless, the patient’s advanced oncological burden, terminal clinical status and concomitant significant aortic regurgitation made eligibility for any transcatheter tricuspid strategy—including an investigational one—highly unlikely.

The anatomical basis for the loss of suitability for T-TEER merits emphasis. Successful edge-to-edge repair depends on favorable leaflet anatomy, sufficient graspable tissue and a limited coaptation gap. Large coaptation gaps are among the strongest predictors of suboptimal repair: a gap of approximately 7 mm best discriminates procedural success, with success rates declining as the gap exceeds 10 mm. These thresholds are predictors of procedural success rather than absolute contraindications; in our patient, the combination of a 17 mm gap, marked tethering, extensive carcinoid remodeling and mild stenosis—rather than the gap alone—placed T-TEER outside the range of feasible options [19,20].

With respect to guidelines, ENETS recommends regular NT-proBNP and echocardiography at approximately 6- to 12-month intervals and has proposed a standardized echocardiographic report for CHD [13,21]; NANETS recommends at least annual echocardiography in patients with markedly elevated serotonin output [22]; and the ESC/EACTS valvular guidelines emphasize follow-up of at least moderate tricuspid regurgitation and hemodynamic assessment of severe regurgitation before intervention [23]. Cohort data report that CHD may appear or progress more than five years after initial evaluation [24]. The variability across protocols reflects a limited evidence base, without randomized comparisons of surveillance intervals.

The importance of timing is illustrated by contrasting published experience. Mohd Nasri et al. described carcinoid heart disease detected 19 years after the initial NET diagnosis yet identified at a still-manageable stage of moderate tricuspid regurgitation, underscoring that recognition—rather than disease duration alone—governs treatability [8]. Conversely, large surgical series report that appropriately selected patients, even those in NYHA class III–IV, can undergo valve replacement with meaningful benefit when referred within the operative window [10,25,26]. In our patient, by contrast, the combination of delayed recognition, advanced valve remodeling and cumulative oncological burden had already closed both the surgical and transcatheter windows by the time of tertiary referral. Patients with carcinoid syndrome and hepatic metastases, elevated 5-HIAA or NT-proBNP, or worsening dyspnea should therefore be reassessed periodically within a multidisciplinary pathway integrating oncology, cardiology and advanced echocardiography [9]. The take-home message is that surveillance is required not merely to detect progression, but to detect it before it passes beyond correctability.

## 4. Conclusions

This case illustrates the progressive evolution of carcinoid heart disease in a patient with a metastatic ileal neuroendocrine tumor and active carcinoid syndrome, ultimately resulting in advanced tricuspid valve involvement with severe regurgitation and mild stenosis. More importantly, it shows that the clinical impact of carcinoid heart disease depends not only on the severity of the valvular lesion, but also on how early its progression is recognized.

A key message of this report is that periodic echocardiographic surveillance should be regarded as an essential component of follow-up in patients with carcinoid syndrome, particularly in the presence of hepatic metastases and persistent biochemical activity. In such patients, cardiac involvement may progress gradually and remain clinically underappreciated until the window for effective intervention is lost.

This case also emphasizes that treatment eligibility is determined by anatomy as much as by symptoms. In our patient, advanced leaflet thickening, tethering, restricted motion, mixed tricuspid dysfunction and a large coaptation gap defined a stage of disease no longer suitable for transcatheter repair, while the overall clinical burden made surgical correction unrealistic. The loss of the therapeutic window was therefore both structural and clinical.

In conclusion, the greatest danger in carcinoid heart disease is not progression alone, but progression beyond correctability. Systematic echocardiographic monitoring, integrated with oncological, biochemical and multidisciplinary reassessment, is essential to identify the optimal moment for referral and to preserve the possibility of intervention before irreversible valvular and hemodynamic deterioration occurs.

## Figures and Tables

**Figure 1:**
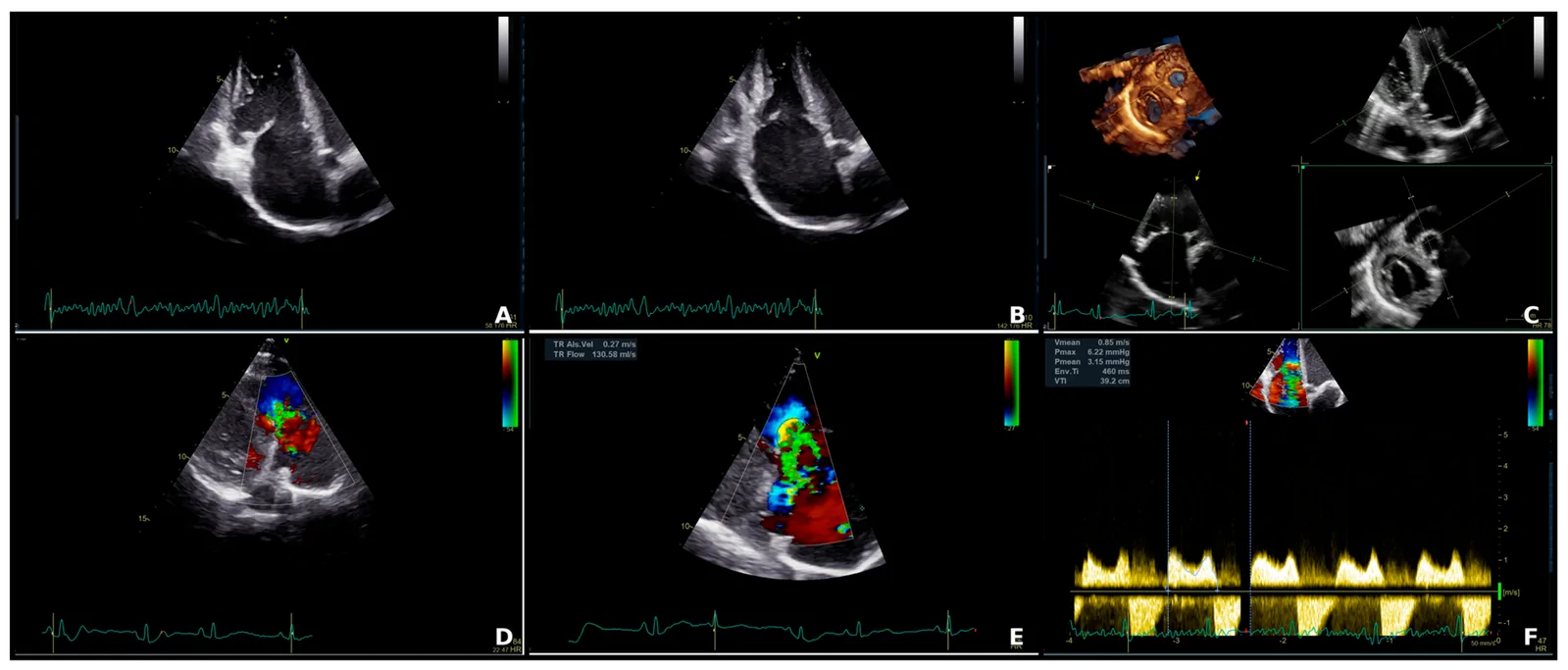
Transthoracic echocardiographic aspects of mixed carcinoid tricuspid valve disease. (**A**) Two-dimensional apical right-heart-focused diastolic frame—thickened, tethered tricuspid leaflets with severely restricted motion. (**B**) Systolic frame showing limited leaflet excursion consistent with carcinoid fibrotic remodeling, resulting in a restrictive tricuspid “tube” appearance. (**C**) Three-dimensional multiplane reconstruction (flexi-slice) of the tricuspid orifice with a planimetric area of 1.7 cm^2^. (**D**) Parasternal right ventricular inflow view demonstrating severe tricuspid regurgitation with a broad regurgitant jet filling the enlarged right atrium. (**E**) Color Doppler with proximal isovelocity surface area (PISA) radius 0.9 cm, aliasing velocity 0.27 m/s, flow rate 130 mL/s, consistent with severe tricuspid regurgitation (EROA 0.6 cm^2^). (**F**) Continuous-wave Doppler across the tricuspid valve demonstrating the stenotic component: peak velocity 1.25 m/s, peak/mean gradient 6.2/3.1 mmHg, confirming mixed valvar disease.

**Table 1:** Timeline of oncological management and cardiac assessment. The contrast between frequent oncological evaluation and the near-absence of structured cardiac assessment before 2025 illustrates the missed surveillance window; cardiac findings are shaded.

Date	Oncological Course	Biomarkers	Cardiac Assessment
December 2019	Ileal NET G2 (Ki-67 up to 5%, pT4 pN1 cM1); segmental ileal + mesenteric nodal resection; hepatic metastases on CT	—	None
June 2020	Abdominal MRI: multiple hepatic metastases (up to 24 mm)	—	None
July 2020	Octreotide 30 mg started	Serotonin 887 µg/L; 5-HIAA 2 mg/24 h; CgA 89 µg/L	None
January 2021	Patient-initiated interruption (symptom resolution)	Serotonin > 1000 µg/L; 5-HIAA 5.3 mg/24 h; CgA 147 µg/L	None
February–August 2021	Lanreotide 120 mg (paused August 2021)	—	None
2022	CT (February, December): slowly progressive hepatic lesions	—	None
August–December 2023	Symptomatic recurrence; progression on CT and Octreoscan; lanreotide resumed	Serotonin 1558 µg/L; 5-HIAA 110 mg/24 h; CgA 1272 ng/mL (September 2023)	None
December 2023	SSTR scintigraphy: Krenning grade 3	—	None
March–August 2024	Lanreotide, then octreotide 30 mg, then discontinued (side effects)	—	None
August 2024–April 2025	Everolimus	—	None
May 2025	First 177Lu-DOTATOC PRRT (7.5 GBq); progressive hepatic burden; peritoneal carcinosis	CgA markedly elevated	First documented cardiac note: moderate tricuspid regurgitation, “under cardiological surveillance”
2025	—	NT-proBNP elevated	TTE: NYHA II HF; LVEF 36%; severe primary tricuspid regurgitation; moderate aortic regurgitation; PFO
December 2025	Ongoing metastatic disease	—	Cardiac MRI: LVEF 26%; non-dilated LV, global hypokinesia; no edema/LGE (ECV 24%); severe TR (regurgitant fraction 51%); RA dilatation; interatrial septal aneurysm
February 2026	Referred for evaluation for transcatheter tricuspid repair	NT-proBNP 4322 pg/mL; macrocytic anemia; cholestatic liver injury	TTE: mixed carcinoid tricuspid disease—severe regurgitation + mild stenosis; coaptation gap 17 mm; leaflet thickening/tethering; gradient 6.2/3.1 mmHg; RV–RA gradient 47 mmHg; 3D orifice 1.7 cm^2^; LVEF 37%; NYHA class IV; unsuitable for T-TEER
2026 (exact date not confirmed)	Death	—	—

—, not available; None, no cardiac assessment documented.

## Data Availability

The data presented in this study are available on request from the corresponding author due to patient-privacy restrictions and medical confidentiality.

## Abbreviations

CHD, carcinoid heart disease; CMR, cardiac magnetic resonance; ECV, extracellular volume; EROA, effective regurgitant orifice area; FAC, fractional area change; 5-HIAA, 5-hydroxyindoleacetic acid; LGE, late gadolinium enhancement; LVEF, left ventricular ejection fraction; NET, neuroendocrine tumor; NT-proBNP, N-terminal pro B-type natriuretic peptide; NYHA, New York Heart Association; PASP, pulmonary artery systolic pressure; PISA, proximal isovelocity surface area; PRRT, peptide receptor radionuclide therapy; TAPSE, tricuspid annular plane systolic excursion; TR, tricuspid regurgitation; T-TEER, tricuspid transcatheter edge-to-edge repair; TTVR, transcatheter tricuspid valve replacement.
